# Potent anti-tumor immunostimulatory biocompatible nanohydrogel made from DNA

**DOI:** 10.1186/s11671-019-3032-9

**Published:** 2019-06-26

**Authors:** Jiana Jiang, Xianming Kong, Yuexia Xie, Hanbing Zou, Qianyun Tang, Ding Ma, Xue Zhao, Xiaozhen He, Anyue Xia, Peifeng Liu

**Affiliations:** 1 Central Laboratory, Renji Hospital, School of Medicine, Shanghai Jiao Tong University, Shanghai, 200032 People’s Republic of China; 2 State Key Laboratory of Oncogenes and Related Genes, Shanghai Cancer Institute, Renji Hospital, School of Medicine, Shanghai Jiao Tong University, Shanghai, 200032 People’s Republic of China; 3 Xinjiang Tumor Hospital affiliated to Xinjiang Medical University, Urumqi, Xinjiang 830011 People’s Republic of China

**Keywords:** CpG, Nanohydrogel, Immunotherapy, Macrophage, Glioma

## Abstract

Unmethylated CpG oligodeoxynucleotides are potent immunostimulatory motifs in activating both innate and acquired immune system by inducing Th1 type antigen-specific T cell responses, but their instability in serum greatly influences their immunostimulant efficiency. Here, we constructed a novel immuno-DNA nanohydrogels consisting of tandem repeat sequences of CpG units named CpG-MCA nanohydrogels through multi-primed chain amplification. CpG-MCA nanohydrogels were proved to resist degradation and increase the proliferation and migration of murine macrophage-like RAW264.7 cells. Furthermore, CpG-MCA nanohydrogels effectively induced high expression of tumor necrosis factor-α and interleukin-6, and remarkably inhibited the proliferation of U251 cells, suggesting that CpG-MCA nanohydrogels are expected to be employed as the potent anti-cancer immunostimulant.

**Electronic supplementary material:**

The online version of this article (10.1186/s11671-019-3032-9) contains supplementary material, which is available to authorized users.

## Introduction

Bacterial DNA containing unmethylated CpG motifs are extremely promising vaccine adjuvants, anti-allergens, and immunoprotective and anticancer agents [1]; it could be recognized by receptors on endosomal inside immune system cells, such as dendritic cells (DC), macrophages, T cells, natural killer (NK) cells, and NKT cells [2]. These innate immune cells are able to respond to unmethylated CpG motifs through the recognition of pathogen-associated molecular patterns (PAMPs) to pathogen-specific molecules in the pathogenic microorganism. It had been confirmed that CpG oligonucleotide (CpG-ODN) could be recognized by Toll-like receptor 9 (TLR9) [3] and induced a Th1-type immune response through Myd88-dependent signaling pathway [4]. However, these drawbacks of CpG-ODN impeded its clinical application owing to its prematuration by protein adsorption, digestion by endonucleases in serum [5], and instability in vivo. These problems are expected to resolve by encapsulating single-strand (ss) nucleic acid into a delivery carrier or making it self-assemble into a nanostructure that improves its stability in vivo as well as more efficient internalization ratio to innate immune cells. Currently, various carriers such as cationic polymer polyethylenimines (PEI) [6], liposomes [7, 8], and microparticles [9] were utilized to the delivery of CpG-ODN; there are still some drawbacks that remain to be improved, such as its cytotoxicity, limited loading rate and etc.

**Fig. 1:**
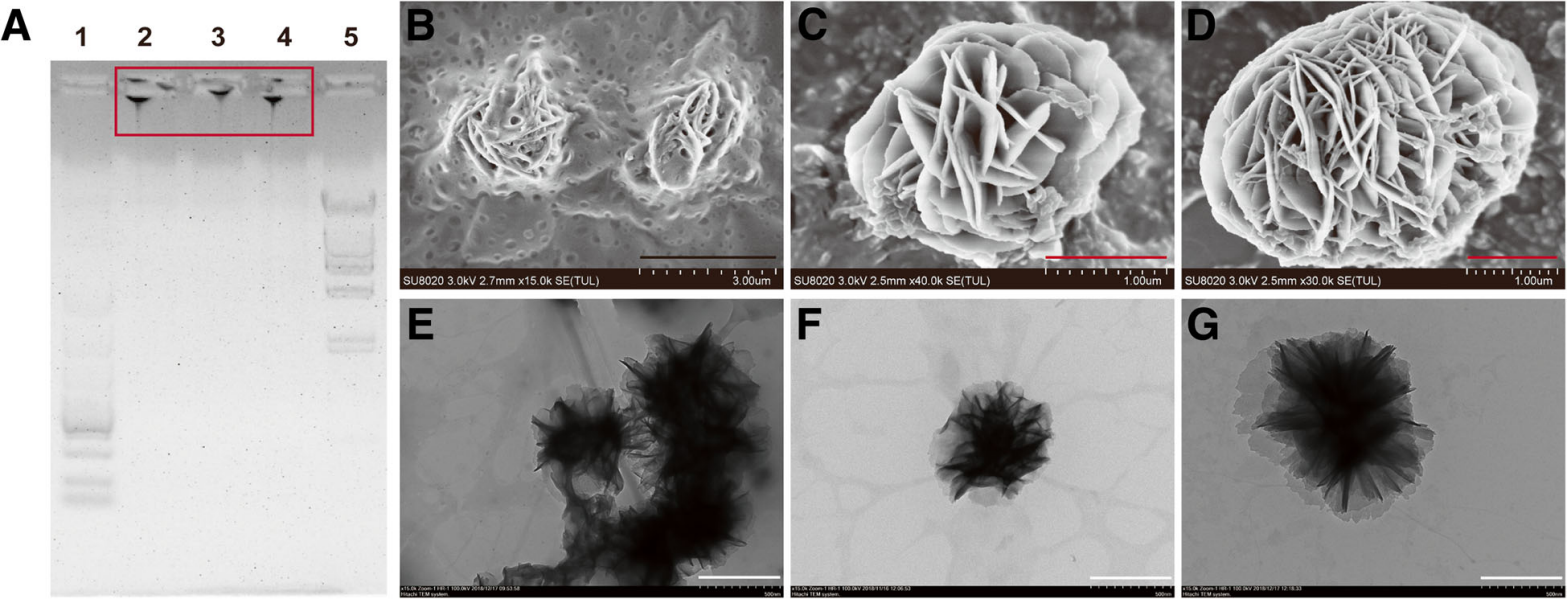
The images of CpG-MCA gels and CpG-RCA gel. **a** Agarose gel electrophoresis image of CpG-RCA gel (R12) and CpG-MCA gels (R4M4 and R4M8). Lane 1, DNA MW standard marker λ-Hind III digest; lane 2, R12; lane 3, R4M4; lane 4, R4M8, lane 5, DL5000 DNA marker. SEM images of R12 (**b**), R4M4 (**c**), and R4M8 (**d**). TEM images of R12 (**e**), R4M4 (**f**), and R4M8 (**g**). Black scale bar is 3 μm; red scale bar is 1 μm; white scale bar is 500 nm

**Fig. 2:**
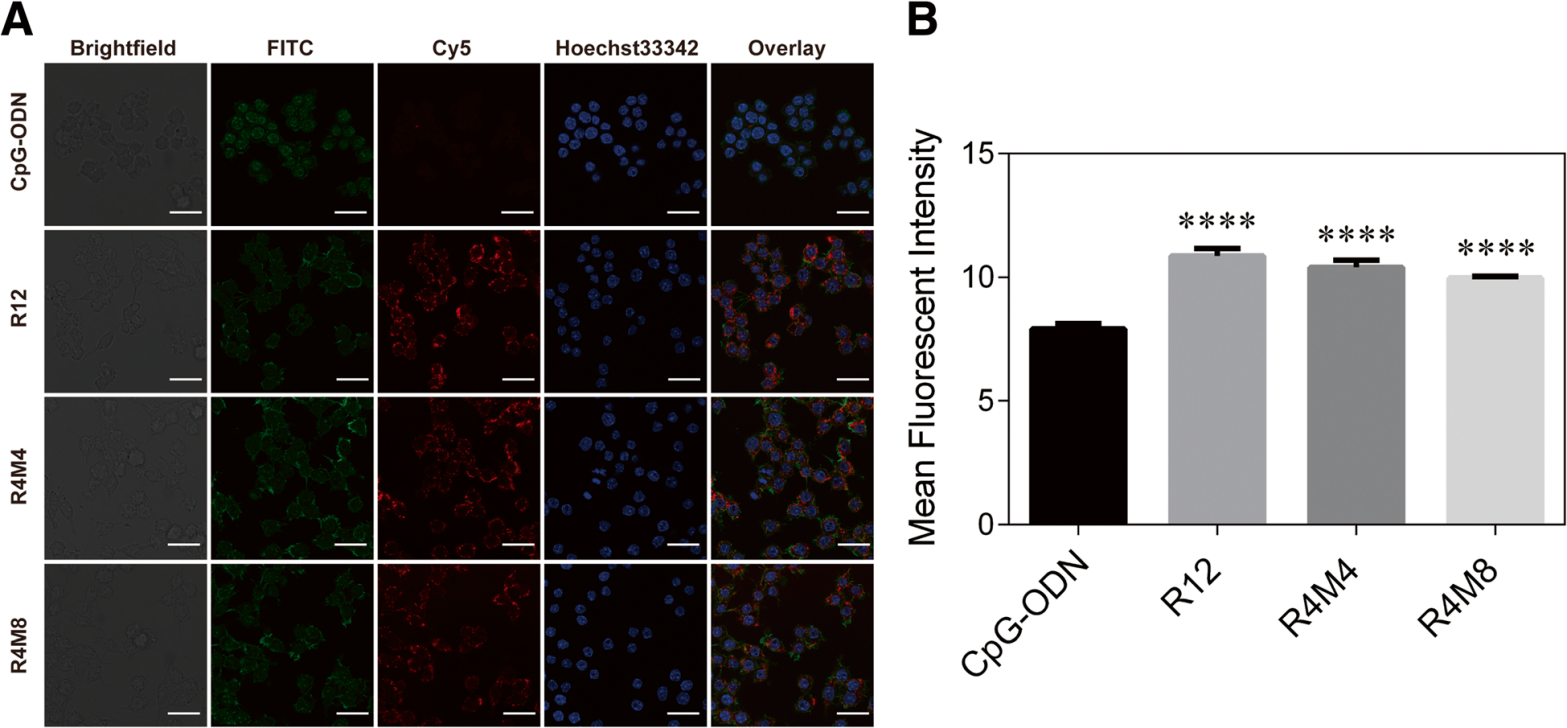
The images of confocal microscope and mean fluorescent intensity in RAW 264.7 cells treated with CpG-ODN, CpG-RCA gel (R12), and CpG-MCA gels (R4M4 and R4M8) (100 nM CpG equivalents). CpG-MCA gels and CpG-RCA gel were labeled with Cy5 (red) through adding Cy5-dCTP for their cellular uptake. Cy5-labeled CpG-ODN was used as a control group. **a** Confocal microscopy images of CpG-RCA gel and CpG-MCA gels by RAW 264.7 cells after incubated for 2 h. **b** The mean fluorescent intensity of Cy5 in RAW264.7 cells. Results are expressed as the mean ± SD of three independent experiments. ****P < 0.0001

DNA materials that are made up of nucleic acid show great potential to be used as carriers of delivering CpG-ODN. Compared with ssDNA, DNA materials with two-dimension or three-dimension structure exhibit different properties, such as easy penetration of cell membranes and stimulation of macrophages to secrete cytokines [10]. X-shaped DNA was utilized to deliver CpG motifs and successfully increase the immunostimulatory activity of CpG-ODN by increased cellular uptake, whereas the increased cellular uptake is partly due to the X-shape structure [11]. Similarly, a three-dimension DNA tetrahedral that can be self-assembled into nanostructures with uniform sizes was non-invasively and efficiently entered into RAW264.7 cells to function. What is more, such tetrahedral was proved to be mechanically stable and non-cytotoxic according to the research [12]. Recently, CpG-RCA hydrogel (CpG-RCA gel) with a nanoflower structure prepared through rolling circle amplification (RCA) had been demonstrated that it was able to deliver immune-stimulating signal, resist nuclease degradation, increase the secretion of immune cytokines, and inhibit the proliferation of human acute lymphocytic leukemia T lymphocyte (CCRF-CEM) cells [13]. These above results suggested that the shape and structure of DNA materials played an important role in enhancing cellular uptake and increasing immune stimulation efficiency. Because CCRF-CEM cells are from human T lymphoblast leukemia, a type of hematologic malignancy, it was different from a hematologic malignant tumor; solid tumors often surround with an immunosuppressive microenvironment that can impede valid anti-tumor immunity [14]. For this, we constructed a DNA immunostimulant containing much more copies of CpG-ODN through multi-primed chain amplification (MCA) [15] rather than RCA [16]. Taking advantage of the principle of complementary base pairing, CpG-involved primer and specifically designed template sequence were mixed to ligase and extended by phi29 polymerase in the presence of free dNTPs. RCA (R) or MCA (M) reacted for *x* or *y* hours to be separately represented as Rx or My (Fig. 1); the products were identified by agarose gel electrophoresis (Fig. 2a). Based on the MCA reaction, the obtained products we called CpG-MCA hydrogels (CpG-MCA gels) possessing hundreds or thousands of tandem CpGs due to the great increase of CpG motif copies. CpG-MCA gels were also a potent immunostimulant that significantly increased the secretion of cytokines from RAW264.7 cells and effectively inhibited the proliferation of human glioma U251 cell lines. We expected that this study is conductive to a novel nanohydrogel immunostimulant based on DNA materials and promoted its application for tumor immunotherapy [17].

## Methods/Experimental

### Materials

All the oligonucleotides were purchased from Sangon Biotech (Shanghai) Co., Ltd. and purified by high-performance liquid chromatography. dNTP sets (100 mM each), phi29 DNA polymerase (10 U/μL), and 10× phi29 DNA polymerase reaction buffer (330 mM Tris-acetate (pH 7.9 at 37 °C), 100 mM Mg acetate, 660 mM K acetate, 1% (*v*/*v*) Tween 20, 10 mM DTT) were purchased from Thermo Fisher Scientific (Waltham, MA, USA). T4 DNA ligase (400 U/μL), 5′-triphosadenine (ATP), and 10× T4 DNA ligase reaction buffer (50 mM Tris-HCl, 10 mM MgCl_2_, 1 mM ATP, 10 mM DTT, pH 7.5 at 25 °C) were purchased from New England Biolabs, Inc. (Ipswich, MA). Cyanine 5-dCTP was purchased from PerkinElmer, Inc. (Waltham, MA, USA). Amicon Ultra-0.5 centrifugal filter devices were purchased from Merck KGaA (Darmstadt, Germany). The water used in this paper was purified by Millipore Synergy UV Ultrapure water purification system. Gibco fetal bovine serum (FBS), Dulbecco’s modified Eagle medium (DMEM, with high glucose, L-glutamine, phenol red, sodium pyruvate, without HEPES), Trypsin-EDTA (0.25%), and penicillin (10000 U/mL)-streptomycin (10000 μg/mL) were purchased from Thermo Fisher Scientific (Waltham, MA, USA). ELISA kits were purchased from R&D Systems, Inc. Cell Counting Kit-8 (CCK-8) was purchased from Dojindo (Kumamoto, Japan). The mouse macrophage-like cell line (RAW264.7 cells) was obtained from the cell bank of Chinese Academy of Sciences (Shanghai, China). The human glioma cell lines (U251 cells) were obtained from the cell bank of Chinese Academy of Sciences (Shanghai, China).

### Preparation of Circular DNA Templates

Long single-stranded DNA with a phosphorylated group at 5′ end with equal ratio CpG-ODN primers (primer 1) were mixed in 1× phi29 reaction buffer and annealed at 95 °C for 5 min and slowly cooled to 4 °C at a rate of − 1 °C/s using a thermal cycler (Bio-Rad T100, Germany). Following the annealing, 20 U/μL T4 DNA ligase with ATP and T4 DNA ligase reaction buffer was added and incubated at 4 °C overnight. Enzymes were inactive at 75 °C for 10 min.

### Preparation of CpG-MCA Gels and CpG-RCA Gel

A circular DNA template (10 μL) was mixed with 1× phi29 DNA polymerase reaction buffer, 4 mM dNTP for each, and 5 U phi29 DNA polymerase and sterile DDW was added, 50 μL in total. The mixture was incubated at 30 °C with shaking for 12 h (R12). For the MCA gel formation, after 4 h RCA reaction, 500 pM of primer 2 and primer 3 were then added into the resultant mixture respectively to be incubated for the rest hours at 30 °C without adding additional reagents (R4M4 and R4M8). The phi29 polymerase was inactivated at 65 °C for 10 min. The CpG-RCA gel and CpG-MCA gels were purified by ultrafiltration.

### Concentration of CpG-MCA Gels and CpG-RCA Gel

The concentration of CpG-MCA gels and CpG-RCA gel was measured based on the number of CpG copies involved in all hydrogels in the treatment group. Since dNTP added into the reaction system was 4 mM for each, one circular template contained 81 nucleotides and 1 copy of CpG. If Abs is the absorbance of dNTP at 260 nm and *ε* is the extinction coefficient of dNTP at 260 nm, then the consumed dNTP in the reaction could be measured and total copies of CpG calculated by the following equation: CpG copies = (4 mM × 4 − Abs/*ε* × 1,000,000)/81 [13]. Abs and *ε* of dNTP were measured by NanoDrop 2000c.

### Agarose Gel Electrophoresis

Agarose gel electrophoresis was used to evaluate the formation and degradation of CpG-MCA gels and the formation of a circular template. Hydrogels were run on 1% agarose gel at 100 V for 60 min, and the circular template was run on 3% agarose gel at 100 V for 60 min.

### Characterization of CpG-MCA Gels and CpG-RCA Gel

Transmission electron microscopy (TEM, Hitachi HT7700, Japan) was employed to characterize the CpG-MCA gels’ inner structure and approximate size. CpG-MCA gels were examined by ultrasound for 30 min before being deposited on copper and dried. Tests were carried out at the center lab of Renji Hospital. Scanning electron microscopy (SEM, Hitachi SU8020, Japan) was used to obtain the morphology of the CpG-MCA gels. CpG-MCA gels were examined by ultrasound for 30 min before being deposited on a clean silicon wafer, and the sample was metal-coated with Au.

### Confocal Microscopic Imaging

Cell uptake was imaged by a Leica confocal microscope. RAW264.7 cells were seeded on a confocal petri dish at a density of 2 × 10^5^ cells/mL. After washing twice with phosphate buffer (PBS), cells were incubated with 100 nM Cy5-labeled CpG-ODN, CpG-RCA gel, and CpG-MCA gels in fresh DMEM medium for 2 h at 37 °. Cells were then washed three times with PBS and fixed with 4% paraformaldehyde for 30 min, then cells were stained with FITC-phalloidin and Hoechst 33342. All images were taken using a Leica laser confocal microscope. The semiquantitative of mean fluorescent intensity was calculated by Image J, a Java-based application for analyzing images.

### ELISA Assay

RAW264.7 cells were seeded at a density of 7 × 10^4^ cells/mL in a 24-well plate cultured for 24 h before use. The cells were incubated at the presence of CpG-MCA gels and other groups at 37 °C for 8 h for TNF-α and 24 h for IL-6; the supernatants were collected. The levels of cytokines in the supernatants were detected by enzyme-linked immunosorbent assay (ELISA) following protocols suggested by the manufacturer.

### Gene Expression Assay

Gene expression levels were assayed by quantitative real-time PCR (Q-PCR). RAW264.7 cells were seeded at a density of 1 × 10^6^ cells/mL in a 6-well plate cultured for 24 h before use. Cells were incubated at the presence of CpG-MCA gels and other groups at 37 °C for 2 h for TNF-α and TLR9 and 8 h for IL-6 and others. Isolation and purification of mRNA were performed using TRIzol Reagent (Thermo Fisher Scientific). Extracted mRNA was quantified by NanoDrop 2000c. One microgram of total RNA was reverse-transcribed using PrimeScript RT reagent Kit with gDNA Eraser (Takara Bio Inc.); amplification was performed in a total reaction volume of 20 μL, using TB Green Premix Ex Taq II (Takara Bio Inc) according to the manufacturer’s instructions. Primers for genes are as follows: GAPDH: F: AGGTCGGTGTGAACGGATTTG; R: TGTAGACCATGTAGTTGAGGTCA; TLR9: F: ATGGTTCTCCGTCGAAGGACT; R: GAGGCTTCAGCTCACAGGG; TNF-α: F: GACGTGGAACTGGCAGAAGAG; R: TTGGTGGTTTGTGAGTGTGAG; IL-6: F: CCAAGAGGTGAGTGCTTCCC; R: CTGTTGTTCAGACTCTCTCCCT; CD86: F: GAGCTGGTAGTATTTTGGCAGG; R: GGCCCAGGTACTTGGCATT; CD206(MRC1):F: CTCTGTTCAGCTATTGGACGC; R: CGGAATTTCTGGGATTCAGCTTC.

### Scratch Wound Migration Assay

RAW264.7 cells were seeded 70 μL at a density of 5 × 10^5^ cells/mL in culture inserts for 24 h before use. Then, the inserts were removed carefully and cells were washed twice before being treated with each group in a fresh medium. Photos were collected in 0 h, 6 h, and 24 h; the wound area was measured by ImageJ. Each group had three repeats and the experiment was repeated for three times.

### Cytotoxicity Assay

Cytotoxicity was assayed using CCK8. RAW264.7 cells were seeded in 96-well plates and treated with each group for 24 h. Then, 10 μL of CCK8 solution was added to each well, followed by 1–2 h of incubation at 37 °C. The absorbance was measured at 450 nm, each group had three repeats, and the experiment was repeated three times.

### U251 Cells Co-cultured with RAW264.7 Cells

RAW264.7 cells were seeded on the upper chambers and U251 cells were seeded on the lower chambers separately and incubated for 24 h prior to treatment. After washing with PBS three times, 1 μM GpC-ODN, CpG-ODN, CpG-RCA gel, and CpG-MCA gels in the fresh medium were added into both upper and lower chambers for the indicated time. The U251 cells on lower chambers were collected and conducted the plate cloning experiment.

### Plate Clone Formation Assay

The effect on U251 cells proliferation of co-cultured with RAW264.7 cells was analyzed with plate cloning experiments. U251 cells on the lower chambers were collected and diluted with multiple proportions in DMEM containing 15% FBS on a final number of 200 cells/well on a 6-well culture plate and continued to culture for 2 weeks until the clone clusters could be observed by naked eyes (more than 50 cells/clone). After gently washing with PBS, cells were fixed with 4% paraformaldehyde for 30 min, then stained with crystal violet for 1 h. Clusters containing more than 50 cells would be included in the count as a successful clone formation. The experiments were repeated three times and each experiment had three repeat wells: clone formation rate = (clone formation number/inoculated cell number) × 100%.

### Stability of CpG-MCA Gels and CpG-RCA Gel

Freeze-dried CpG-MCA gels or CpG-RCA gel was put in 400 μL DMEM containing 10% fetal bovine serum (10% FBS-DMEM) respectively and incubated in 37 °C for 24 h; the DNA concentrations in the supernatant were measured by NanoDrop 2000c after being centrifuged at 1000 rpm for 10 s. The remaining hydrogels were calculated according to the DNA concentrations in the supernatant. The remaining gel = (*m* − *c* × *V*)/*m* × 100%, where *m* is the gel mass we added, *c* is the DNA concentration in the supernatant, and *V* is the volume of supernatant. CpG-MCA gels or CpG-RCA gel was put in 10% FBS-DMEM or PBS respectively and incubated in 37  °C for 12 h or 24 h. After incubating, gels were run on 1% agarose gel at 100 V for 60 min.

### CpG-ODN

Single-strand CpG-ODN was synthesized by Sangon Company (Beijing, China) and purified using high-performance liquid chromatography (HPLC). The CpG-ODN used in this study was CpG-ODN 1668 [18]: 5′-TCCATGACGTTCCTGATGCT, and the non-CpG oligonucleotides were named GpC-ODN [18]: 5′-TCCATGAGCTTCCTGATGCT.

### Statistical Analysis

All data in this study are represented as mean values ± standard deviation (mean ± SD) from two or three independent experiments. Statistical analysis between different groups was performed through a Student’s *t* test. The statistical significance was set at *P* < 0.05 (95% confidence level).

## Results and Discussion

**Scheme 1:**
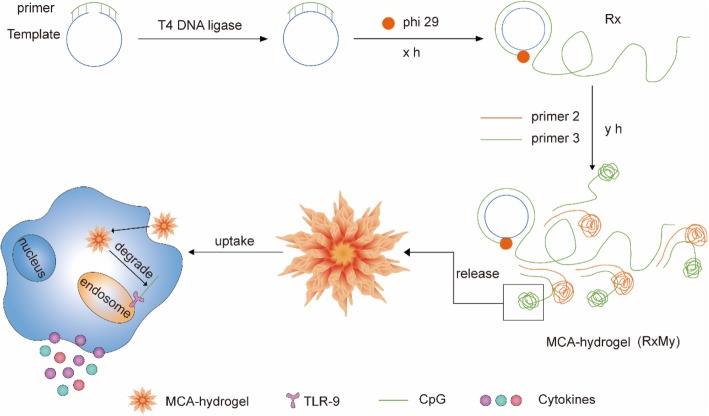
The schematic illustration on the preparation and cellular uptake of CpG-MCA gels and CpG-RCA gel. Long single-stranded DNA with a phosphorylated group at 5' end were mixed with primers composed of CpG motifs were first annealed and ligated by T4 DNA ligase to form a circular template. RCA and MCA reaction was performed by phi29 DNA polymerase to generate a large amount of sequential concatemer DNA and convolve like many nanoflowers. Gels can then be uptake by macrophages and stimulate the secretion of cytokines

The DNA immunostimulant was successfully constructed through MCA reaction [16]. In this reaction, the first and important step before isothermal amplification is the cyclization reaction of a long single-strand (ss) template (Scheme 1). We employed agarose gel electrophoresis to confirm the formation of circular DNA after annealing and ligation with a primer (Additional file 1: Figure S1). The migration distance between the primer and long ssDNA template became shorter since the structure changed after the template becamed a cycle and ligated with primer. The MCA reaction contains a string of repeating units in series and easily led to the appearance of nanoflowers due to the continuous winding (Scheme 1). Agarose gel electrophoresis results demonstrated that the products of RCA reaction (CpG-RCA gel or R12) and MCA reaction (CpG-MCA gel or R4M4/R4M8) were successful obtained (Fig. 1a). CpG-RCA gel and CpG-MCA gels were difficult to migrate through the agarose gel and remained the retention in home position. Both gels were too sticky so they were pulled out as silk when we are pipetting them (Additional file 1: Figure S2A–C). These results suggested that the DNA template is exponentially amplified with free dNTPs to form a hydrogel [15]. It is worth mentioning that the migration distance was of no significant difference between the CpG-RCA gel and CpG-MCA gels. In addition, SEM and TEM results further showed that the diameter of CpG-RCA gel (Fig. 1b, e) and CpG-MCA gels (Fig. 1c, d, f, and g) were ranged from nanoscale to micrometer scale, and exhibited a morphology of nanoflowers. The CpG-ODN need to be internalized into TLR9-positive immune cells and interact with TLR9 that localized in the endosomes inside immune cells to execute its immunostimulatory activity. For this, we used Cy5-labeled CpG-ODN, CpG-RCA gel, and CpG-MCA gels to research their uptake in the RAW264.7 cells using confocal microscopy. As observed in Fig. 2a, Cy5-labeled CpG-MCA gels were effective internalized and distributed in the cytoplasm of RAW264.7 cells. Based on the mean fluorescent intensity measured, the uptake efficiency of CpG-MCA gels was significantly increased (*P* < 0.0001) compared with that of CpG-ODN (Fig. 2b), demonstrating the effective uptake of CpG-MCA gels, which is favorable for exerting stronger immune stimulation. It is reported that the uptake of DNA by mouse macrophage RAW264.7 cells was increased by designing different DNA nanostructures. Such as tetrapod-like structured DNA (tetrapodna), tetrahedral DNA (tetrahedron), and tetragonal DNA (tetragon) [19]. Similarly, X-shaped-DNA, Y-shaped-DNA and X-DNA hydrogel were also proved to increase the uptake of DNA by cells [20, 21]. These results suggested that higher-order structures of DNA were more efficient forms to deliver functionalized DNA fragments. We therefore speculated that the high cellular uptake of CpG-MCA gels derived from the change of CpG-MCA gels shape through gel formation. We then tested the stability of CpG-MCA gels. CpG-MCA gels were incubated at 37 °C for different time in different solutions. Agarose gel electrophoresis results showed that CpG-MCA gels were relatively stable in PBS solution and were degraded partially in medium involved serum (Additional file 1: Figure S3A). CpG-MCA gels still showed like a ladder rather than a single band which suggested an incomplete degradation. We subsequently researched the stability of gels in 10% FBS-DMEM. TEM results confirmed that CpG-MCA gels were partially digested and no longer look like flowers but still hold the shape (Additional file 1: Figure S2D–F). The degradation curve of CpG-MCA gels was plotted by detecting the concentration of DNA in the supernatant within 24 h (Additional file 1: Figure S3B). It was demonstrated that after a incubation for 24 h in 10% FBS-DMEM, R12 retained nearly 80%, while R4M4 and R4M8 remained nearly 85%, which matched the results of the agarose gel electrophoresis. In order to confirm the degradation of gels, CpG-MCA gels were incubated at 37 °C up to 48 h in serum, and the production of degradation was run in agarose gel electrophoresis (Additional file 1: Figure S4). Gels no longer look like ladders since they were digested into pieces by the enzyme in the serum and lost their viscosity and became even single nucleotide pieces, suggesting that CpG-MCA gels are biodegradable as they could be digested at the presence of fetal bovine serum (FBS). Accordingly, CpG-MCA gels effectively resist serum digestion within 24 h and finally degraded completely. The high ability to resist degradation will help to improve its immune-stimulating efficiency; the expression levels of TNF-α and IL-6 in the supernatant of RAW264.7 cells also clearly showed that CpG-MCA gels could effectively stimulate cells to produce immune cytokines.

**Fig. 3:**
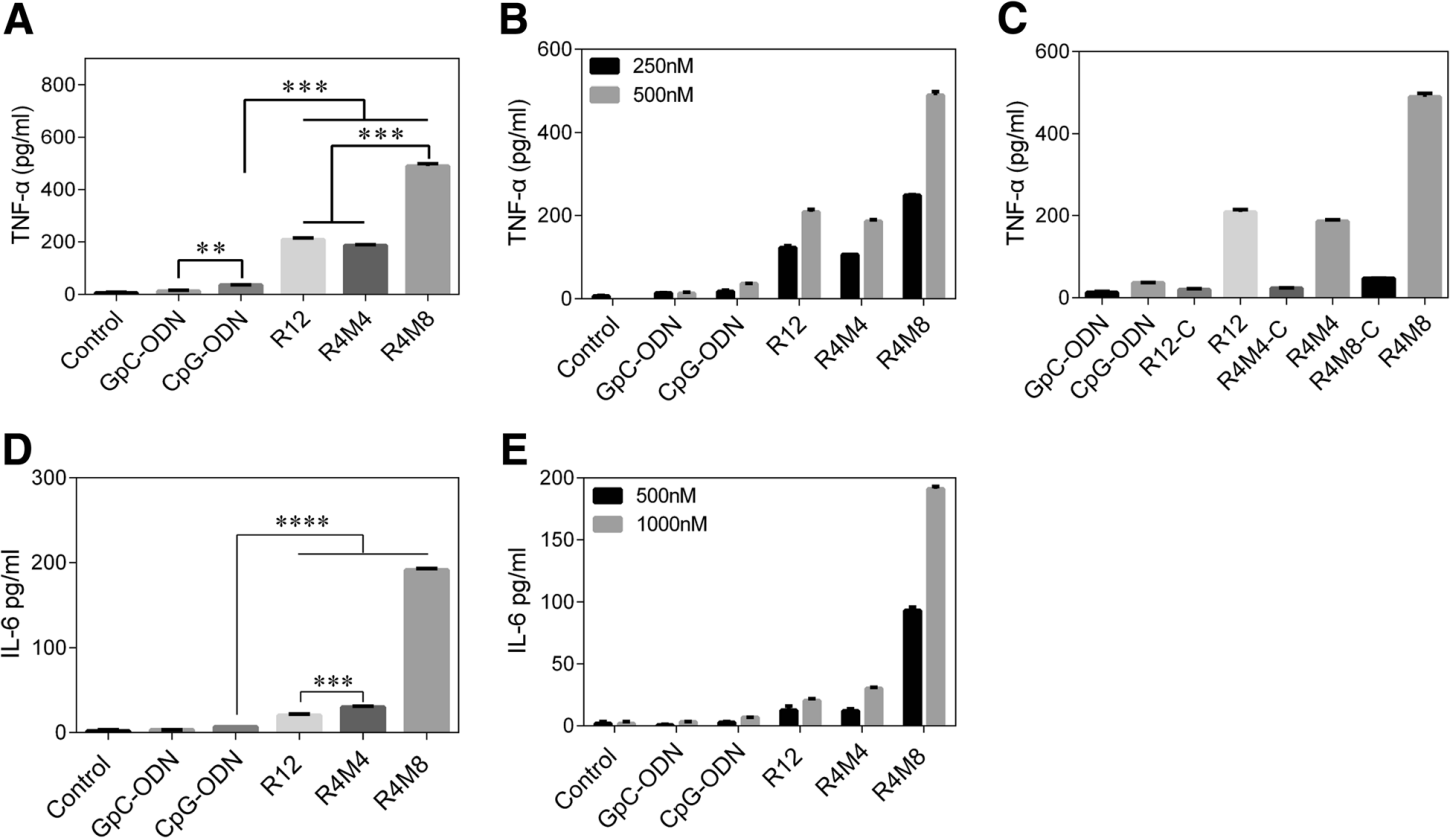
Detection of cytokines released from RAW264.7 cells stimulated with CpG-ODN, CpG-RCA gel, and CpG-MCA gels using ELISA-kit. **a** The secretion of TNF-α from RAW264.7 cells. **b** The secretion of TNF-α from RAW264.7 cells after treated with different concentrations of CpG-MCA gels. **c** Detection of the immuno-stimulating ability of CpG gels and non-CpG gels (R12-C, R4M4-C, and R4M8-C). **d** The secretion of IL-6 from RAW264.7 cells. **e** The secretion of IL-6 from RAW264.7 cells after treated with different concentrations of MCA gels. Results are expressed as the mean ± SD of two independent experiments. **P < 0.01, ***P < 0.001, ****P < 0.0001

The immune-stimulating efficacy of CpG-MCA gels were further evaluated, we treated RAW264.7 macrophages with GpC-ODN (the sequence changed from effect GACGTT to GAGCTT) [22], CpG-ODN, CpG-RCA gel, and CpG-MCA gels and then detected the experssion of TNF-α and IL-6. The concentration of TNF-α in the supernatant of RAW264.7 cells incubated for 8 h with CpG-RCA gel (R12) and CpG-MCA gels (R4M4 and R4M8) elicited much higher level of TNF-α than CpG-ODN in the same treatment concentration (Fig. 3a). For the CpG-MCA gels, R4M8 rather than R4M4 induced higher level of TNF-α secretion (*P* < 0.001), suggesting that TNF-α secretion enhanced as the copies increased. In addition, R4M8 induced a significantly (*P* < 0.001) higher concentration of TNF-α than R12, indicating that CpG-MCA gels are more powerful stimulators than CpG-RCA gels when the total reaction time is equal. The same trend was also found in the detection of IL-6 (Fig. 3d). The secretion of IL-6 stimulated by CpG-MCA gels and CpG-RCA gel was significantly increased. The secretion of IL-6 in the R12, R4M4, and R4M8 groups was 2.97 times (*P* < 0.0001), 4.39 times (*P* < 0.0001), and 27.81 times (*P* < 0.0001) of that in the CpG-ODN group, respectively. The secretion of IL-6 in the R4M8 group was 6.33 times (*P* < 0.0001) of that noted in the R4M4 group and 9.36 times (*P* < 0.001) of that noted in the R12 group. It is worth mentioning that the secretion of IL-6 was of no significant difference among the CpG-ODN, GpC-ODN, and control groups. These results confirmed that a higher efficiency of TNF-α and IL-6 released was observed from RAW264.7 cells treated with CpG-MCA gels rather than from CpG-ODN. Furthermore, the effect of CpG-MCA gels with a different concentration on cytokine secretion was also investigated, and we found that the secretion of TNF-α and IL-6 increased with the concentration of gels (Fig. 3b, e). The immune-stimulating ability result of the non-CpG gels (indicated as R12-C, R4M4-C, and R4M8-C in Fig. 3c) and GpC-ODN demonstrated that they barely induced the secretion of TNF-α (Fig. 3c), indicating that the immune responses induced by CpG-MCA gels result from the CpG motifs.

Next, we tested the mRNA expression of cytokines, cell surface markers, and TLR-9. The TNF-α mRNA in the CpG-ODN group was 1.25 times as the expression detected in the control group, and the fold change in R12, R4M4, and R4M8 groups was 3.28 times (*P* < 0.01), 2.53 times (*P* < 0.05), and 4.57 times (*P* < 0.001) of that tested in the CpG-ODN group separately (Additional file 1: Figure S5A).The mRNA expression of IL-6 in the CpG-ODN group was 1.01 times as much as that in the control group. The IL-6 mRNA expression in the R12, R4M4, and R4M8 groups was 3.87 times (*P* < 0.01), 4.63 times (*P* < 0.05), and 23.04 times (*P* < 0.0001) as much as that in the CpG-ODN group respectively (Additional file 1: Figure S5B).

The mRNA expression of TLR-9, the receptor of CpG inside cells [4], also increased in all groups compared to the control group, but the CpG-MCA gel groups remarkably enhanced the mRNA expression of TLR-9 as compared to the CpG-ODN group (Additional file 1: Figure S5C). In addition to cytokines and TLR9, we also detected costimulatory molecules (CD) CD86 and CD206, which were used to tag pro-inflammatory (M1) and growth-promoting (M2) macrophages, respectively [23]. It was found that there was no significant difference between the CpG-ODN group and the control group in either CD86 or CD206 (Additional file 1: Figure S5D, E). Compared with the CpG-RCA gel group, CD86 in the CpG-MCA gel groups increased in varying degrees, while CD206 decreased in varying degrees, and R4M8 group increased and decreased the most, suggesting that RAW264.7 cells treated with CpG-MCA gels are prone to differentiate into M1 population with CD86 surface marker, which further verified the immune stimulation ability of CpG-MCA gels.

**Fig. 4:**
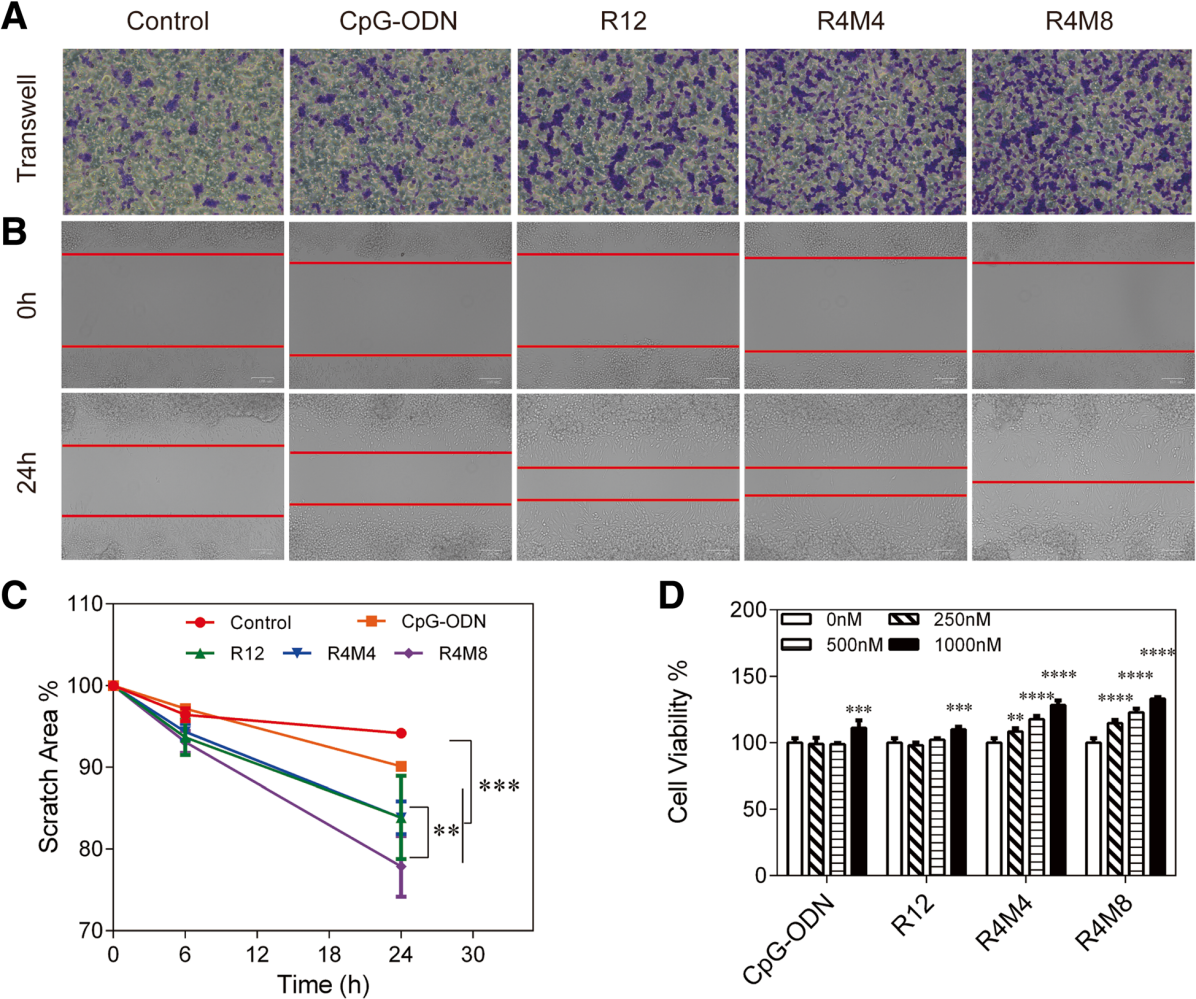
The analysises of migration and cell viability of CpG-ODN, CpG-RCA gel (R12), and CpG-MCA gels (R4M4 and R4M8) in RAW264.7 cells. **a** The migration assay of RAW264.7 cells induced with CpG-ODN, R12, R4M4, and R4M8. **b** The migration assay of RAW264.7 cells stimulated with CpG-ODN, R12, R4M4, and R4M8 for 24 h. **c** The scratch area analysis in the scratch migration experiment. The percentages are calculated as the ratio of the original area. **d** The cell viability of RAW264.7 cells treated with CpG-ODN, R12, R4M4, and R4M8 for 24 h. Results are expressed as the mean ± SD of three independent experiments. **P < 0.01, ***P < 0.001, ****P < 0.0001

The secretion of cytokines from macrophages is extremely important in the response of immune stimulation as well as in the proliferation and migration of macrophages. The stimulation of macrophages with CpG-ODN would increase the production of anti-inflammatory cytokines through TLR9-dependent pathway. The cytokines induced by CpG-ODN increased the migration of macrophages and promoted the proliferation of macrophages by downregulating the expression of a cell cycle negative regulator [24]. CpG-ODN also induced the expression of plasminogen activator inhibitor type-1 (PAI-1) in macrophages, which resulted in an enhanced migration through vitronectin [25]. We also further investigated the ability of CpG-MCA gels to promote macrophages to migrate and evaluate their cytotoxicity. The migration of RAW264.7 cells was induced by CpG-MCA gels in both transwell migration system (Fig. 4a) and scratch wound migration assay (Fig. 4b). RAW264.7 cells were cultured in the presence or absence of CpG-MCA gels and allowed to migrate for 24 h. The migration number of macrophages in the CpG-MCA gel groups was far more than that in the CpG-ODN group (Fig. 4a). Then, we proceeded to the scratch wound migration assay; cells were allowed to migrate for 24 h, and pictures were captured in 0 h, 6 h, and 24 h. The results demonstrated the scratch area was decreased as the wound healed. Migration of macrophages at 6 h showed that the CpG-MCA gels were more effective than the CpG-ODN group as the wound healing ratio was higher, but there is no distinct significance among the R12, R4M4, and R4M8 groups (Additional file 1: Figure S6A, B). And the scratch area at 24 h confirmed that the healing rate of the scratch area in the CpG-ODN group was 1.70 times (*P* < 0.05) of that in the control group, and the healing rate in the R12, R4M4, and R4M8 groups was 1.63 times (*P* < 0.001), 1.63 times (*P* < 0.0001), and 2.23 times (*P* < 0.0001) of that measured in the CpG-ODN group separately. The healing rate in the R4M8 group was 1.36 times (*P* < 0.01) of that in the R12 group. CpG-MCA gels strongly promoted the migration of RAW264.7 cells compared to that treated with the CpG-ODN or control groups (Fig. 4c). In addition, RAW264.7 cells were cultured with a series concentration of gels for 24 h. We found that the CpG-MCA gels exhibited a negligible cytotoxicity to RAW264.7 cells due to the non-toxicity of DNA itself; on the contrary, CpG-MCA gels could stimulate RAW264.7 cell proliferation with a dose-dependent effect, which was a benefit to enhance the production of immune cytokines (Fig. 4d).

**Fig. 5:**
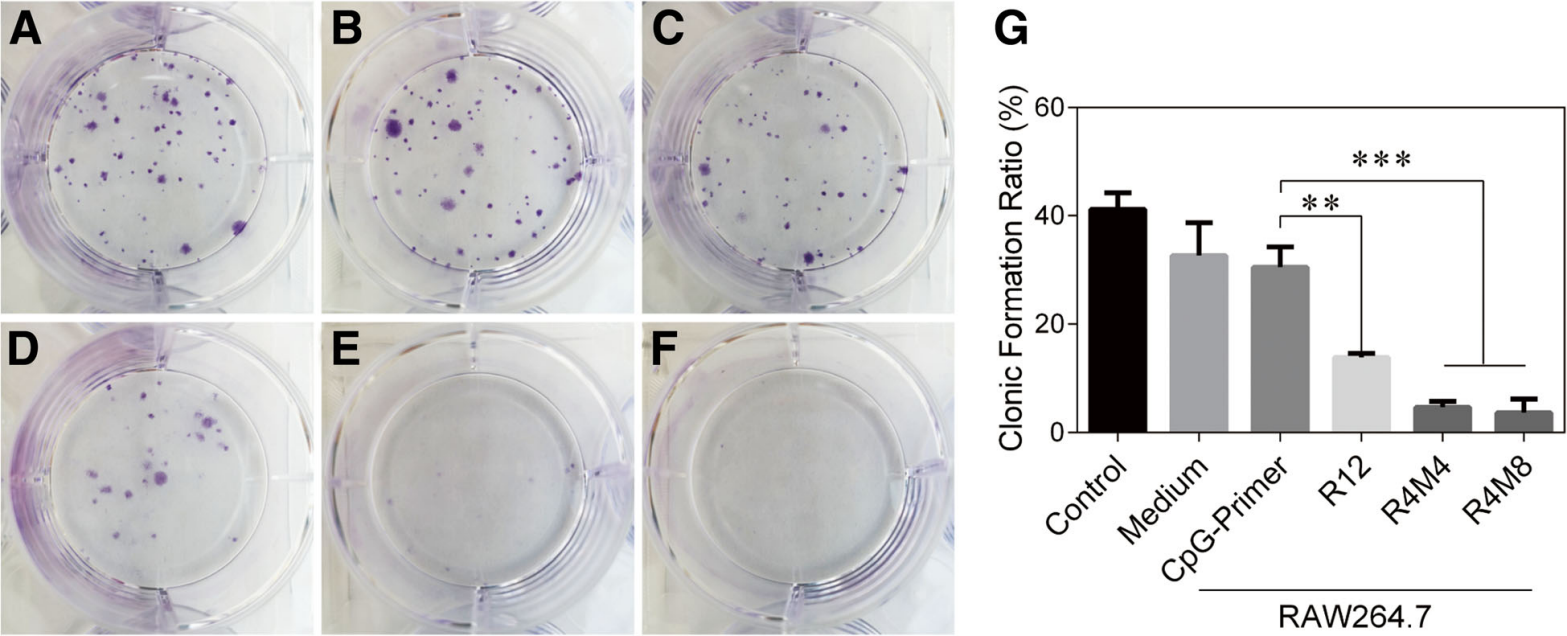
The assessment on the therapeutic effect of immunostimulatory CpG-RCA gel (R12) and CpG-MCA gels (R4M4 and R4M8) on cancer cells in vitro by plate clone formation assay. U251 cells treated with **a** none, **b** RAW264.7 cells, **c** RAW264.7 cells treated with CpG-ODN, **d** RAW264.7 cells treated with R12, **e** RAW264.7 cells treated with R4M4, and **f** RAW264.7 cells treated with R4M8. **g** Results of the proliferation percentage of U251 cells after co-cultured with RAW264.7 cells for 24 h. Results are expressed as the mean ± SD of three independent experiments. **P < 0.01, ***P < 0.001

For further verifying the inhibited efficiency of CpG-MCA gels for the proliferation of solid tumor cells, we estimated the inhibitory effects of CpG-MCA gels as immune stimulators for the U251 human brain glioma cells. The U251 cells was first co-cultured with RAW264.7 macrophages for 24 h, and the clone formation rate was used to check the proliferate ability of the U251 cells. As shown in Fig. 5a–f, the clone formation rate in the CpG-ODN group was 74.1% of that observed in the control group (*P* < 0.05), and the rates in R12, R4M4, and R4M8 groups were 45.4% (*P* < 0.01), 15.3% (*P* < 0.001), and 12.0% (*P* < 0.001) of that in the CpG-ODN group, respectively. The results demonstrated that the U251 cells treated with CpG-MCA gels presented a significantly lower percentage of clone formation rate compared with that treated with the CpG-ODN or control groups (Fig. 5g). It is notable that the trend of inhibitory results was in accordance with the secretion of TNF-α among groups, CpG-MCA gels exhibited a stronger effect on stimulating RAW264.7 cells to secrete cytokines to inhibit proliferation of U251 cells than CpG-ODN. Our research provided preliminary evidence to demonstrate the CpG-MCA gels have the potential to be used as an immunostimulant.

## Conclusions

In summary, we have successfully preparedCpG-MCA nanohydrogels that consist of hundreds of immunostimulatory CpG motifs to effectively deliver immune stimulus signal into cells and significantly induce the expression of immune cytokines. CpG-MCA nanohydrogels exhibited powerful anti-tumor immunity against human glioma cells, demonstrating that CpG-MCA nanohydrogels have the potential to be used as an immunostimulant for the therapy of cancer.

## Additional File

Additional file 1: **Figures S1–S6.** Supplementary Material. (DOCX 2650 kb)

## Data Availability

The datasets used and/or analyzed during the current study are available from the corresponding author on reasonable request.

## Abbreviations

CpG-MCA gels: CpG-MCA hydrogel
CpG-RCA gel: CpG-RCA hydrogel
MCA/M: Multi-primed chain amplification
R12: CpG-RCA gel
R12-C, R4M4-C, R4M8-C: Hydrogels not containing CpG motifs
R4M4, R4M8: CpG-MCA gels
RCA/R: Rolling circle amplification
