# Supplementary material for: Potent anti-tumor immunostimulatory biocompatible nanohydrogel made from DNA
Source: Nanoscale Res Lett. 2019 Jun 26;14:217. doi: 10.1186/s11671-019-3032-9 (PMC6595017; doi:10.1186/s11671-019-3032-9)
Supplement: Supplementary file 1 — Figures S1–S6. Supplementary Material. (DOCX 2650 kb) [file 11671_2019_3032_MOESM1_ESM.docx]

Supplementary information

Potent anti-tumor immunostimulatory biocompatible nanohydrogel made from DNA

Jiana Jiang,‡^a^ Xianming Kong,‡^ac^ Yuexia Xie,^a^ Hanbing Zou,^a^ Qianyun Tang,^b^ Ding Ma,^a^ Xue Zhao,^a^ Xiaozhen He,^a^ Anyue Xia,^a^ and Peifeng Liu*^ab^

*a: Central Laboratory, Renji Hospital, School of Medicine, Shanghai Jiao Tong University.*

*b: State Key Laboratory of Oncogenes and Related Genes, Shanghai Cancer Institute, Renji Hospital, School of Medicine, Shanghai Jiao Tong University, Shanghai 200032, People’s Republic of China.*

*c: Xinjiang Tumor Hospital affiliated to Xinjiang Medical University, Xinjiang 830011, People’s Republic of China.*

*‡ These authors contribute equally to this work.*

** Corresponding author: E-mail: lpf@sjtu.edu.cn; Tel: +86 13512116782.*

**Table S1.** Sequence of Multi-primed chain amplification (MCA).

| 1668 CpG-ODN | TCCATGACGTTCCTGATGCT |
| --- | --- |
| **CpG-MCA** | |
| Template | phos-CGTCATGGAGCTTTTTTTTTTTACGCAGTATTATGGACTGATATAGAATTCTATATATTTTTTTTTTTGCAGCATCAGGAA |
| Primer 1 | GCTCCATGACGTTCCTGATGCTGC |
| Primer 2 | ACGCAGTATTATGGACTG |
| Primer 3 | GCTCCATGACGTTCCTGATGCTGC |
| **GpC-MCA (non-CpG-MCA, MCA-C, control-MCA)** | |
| Template | phos-  GCTCATGGAGCTTTTTTTTTTTACGCAGTATTATGGACTGATATAGAATTCTATATATTTTTTTTTTTGCAGCATCAGGAA |
| Primer 1 | GCTCCATGAGCTTCCTGATGCTGC |
| Primer 2 | ACGCAGTATTATGGACTG |
| Primer 3 | GCTCCATGAGCTTCCTGATGCTGC |


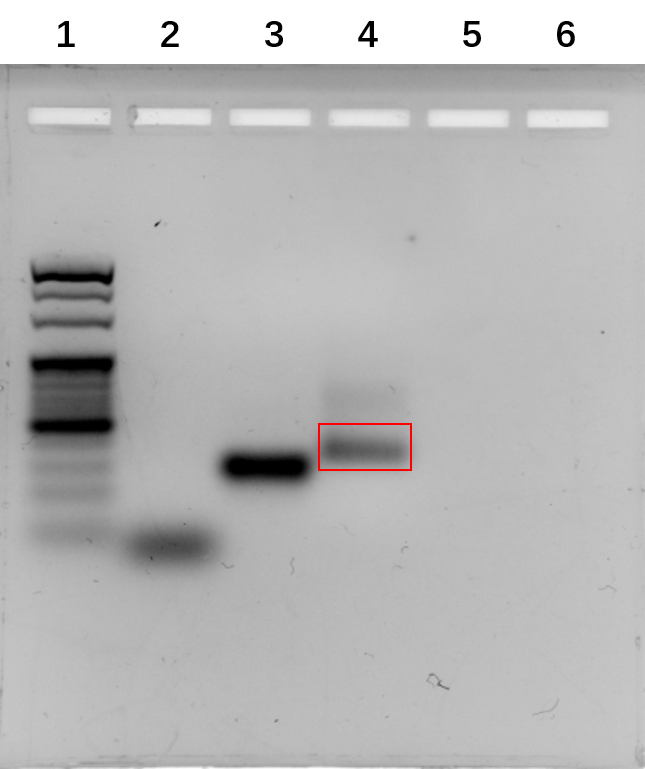


**Figure S1.** Gel electrophoresis image of circular template formation. Gels were run on 3% agarose gel at 100 V for 60 min. (Lane 1) DNA MW standard marker λ-Hind III digest, (lane 2) primer, (lane 3) long single strand DNA, (lane 4) circular template.


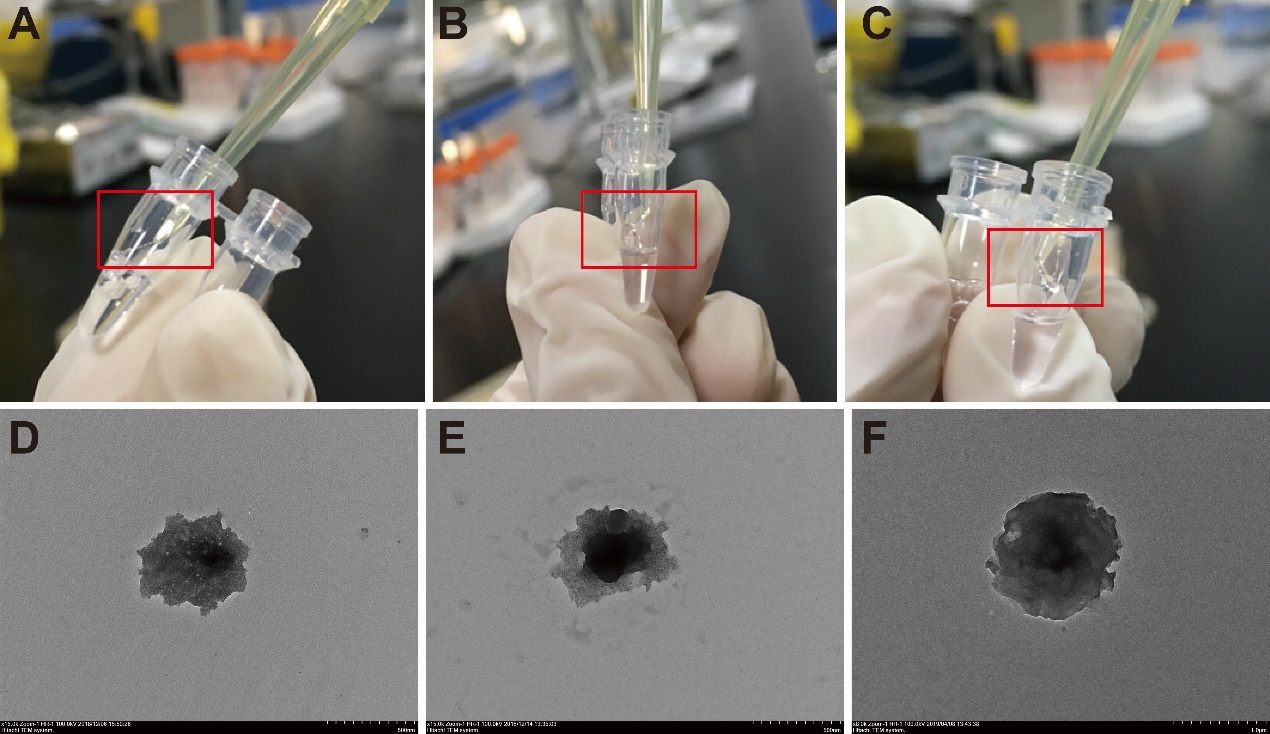


**Figure S2.** Images of CpG-MCA-gels and CpG-RCA-gel. (A) R12, (B) R4M4, (C) R4M8. TEM images of the hydrogels incubated in 10%-FBS-DMEM at 37 °C for 24h. (D) R12, (E) R4M4, (F) R4M8.


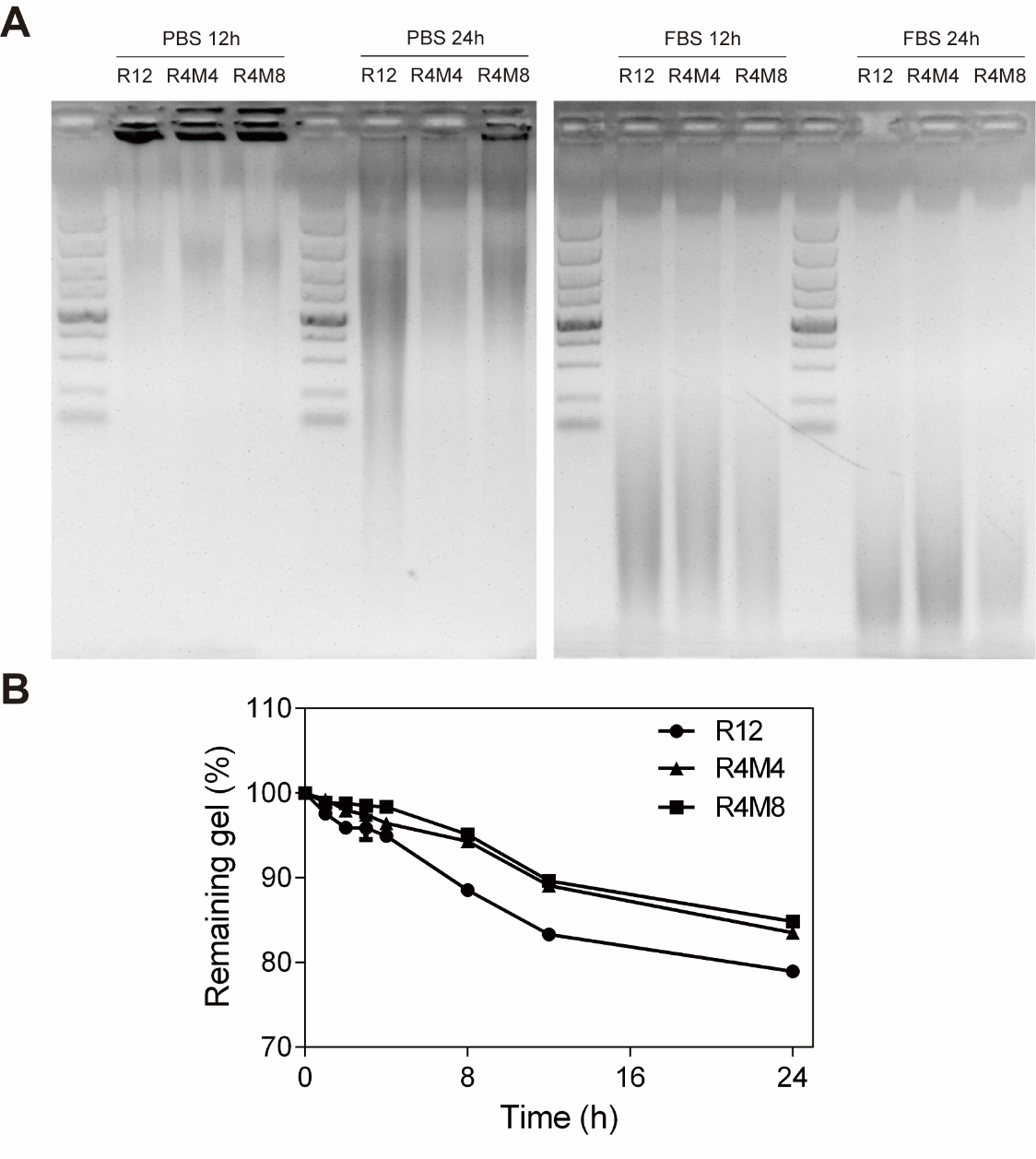


**Figure S3.** The stability assay of CpG-RCA-gel (R12) and CpG-MCA-gels (R4M4 and R4M8) incubated with PBS or 10%-FBS-DMEM respectively at 37 °C for 12h or 24h. (A) Gels were run on 1% agarose gel at 100 V for 60 min. (B) Remaining gels incubated with 10%-FBS-DMEM at 37°C for 24h.


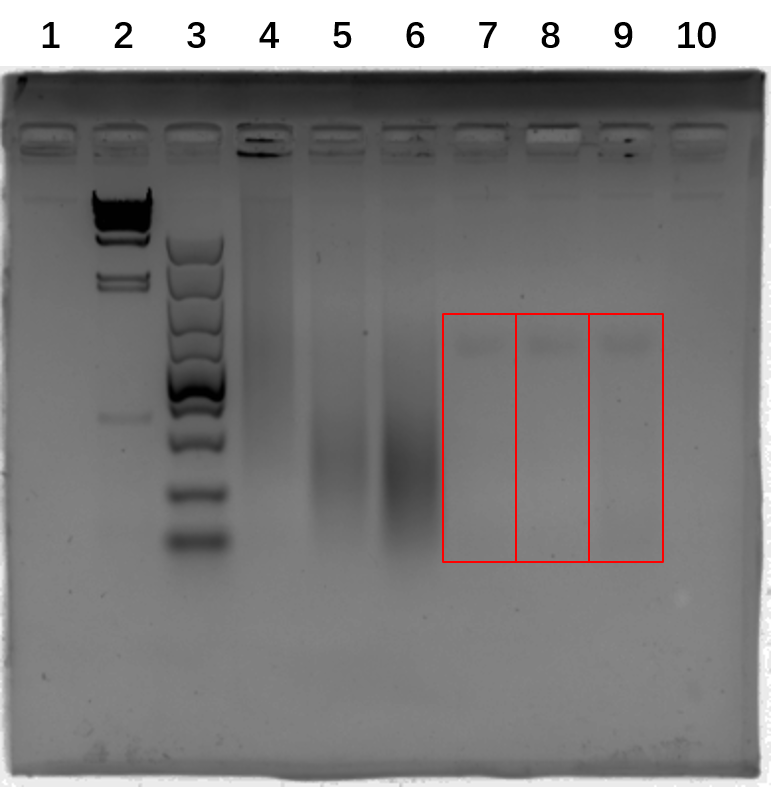


**Figure S4.** The degradation assay of CpG-RCA-gel (R12) and CpG-MCA-gels (R4M4 and R4M8) incubated with 10%-FBS-DMEM at 37 °C for 48 h. Gels were run on 1% agarose gel at 100 V for 60 min. (Lane 1) Empty, (Lane 2) DNA MW standard marker λ-Hind III digest, (lane 3). DL5000 DNA marker, (lane 4) R12, (lane 5) R4M4, (lane 6) R4M8, (lane 7) R12-degradation, (lane 8) R4M4-degradation, (lane 9) R4M8-degradation.


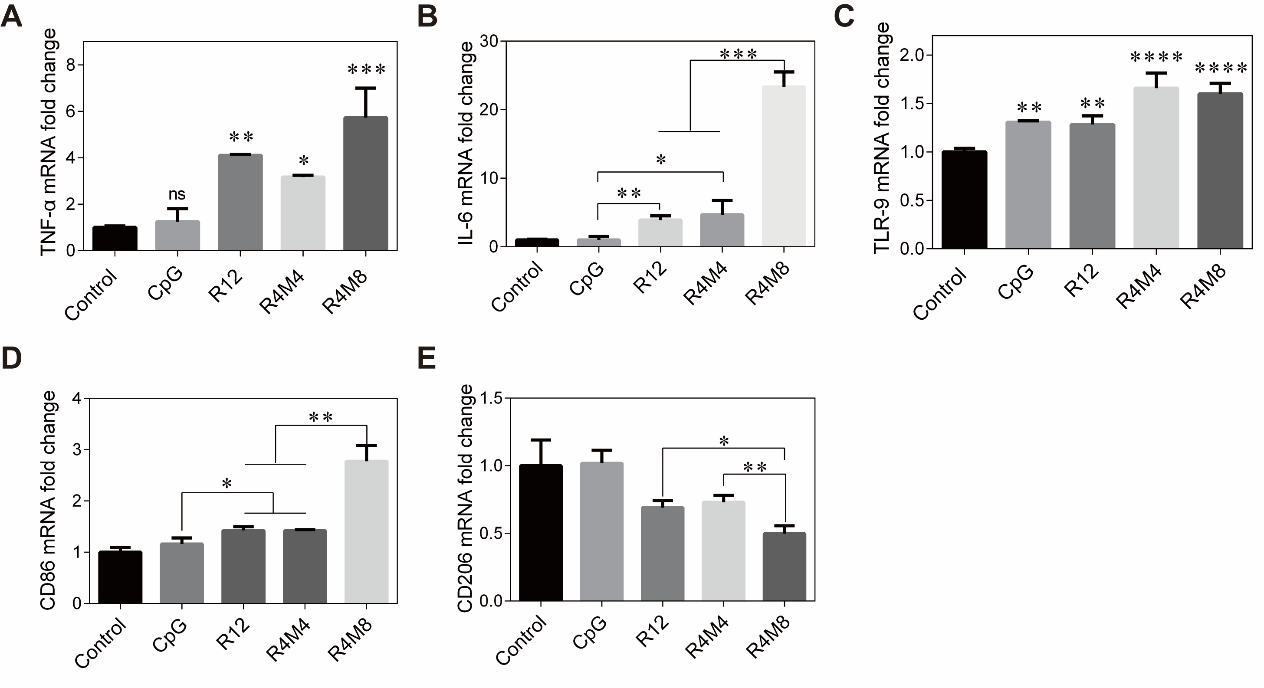


**Figure S5.** The expression of mRNA (A: TNF-α, B: IL-6, C: TLR-9, D: CD86, and E: CD206.) in RAW264.7 cells stimulate by CpG-ODN, CpG-RCA-gel and CpG-MCA-gels. Results are expressed as the mean ± SD of three independent experiments. ns: no significance, * P < 0.05, ** P < 0.01, *** P < 0.001, **** P < 0.0001.


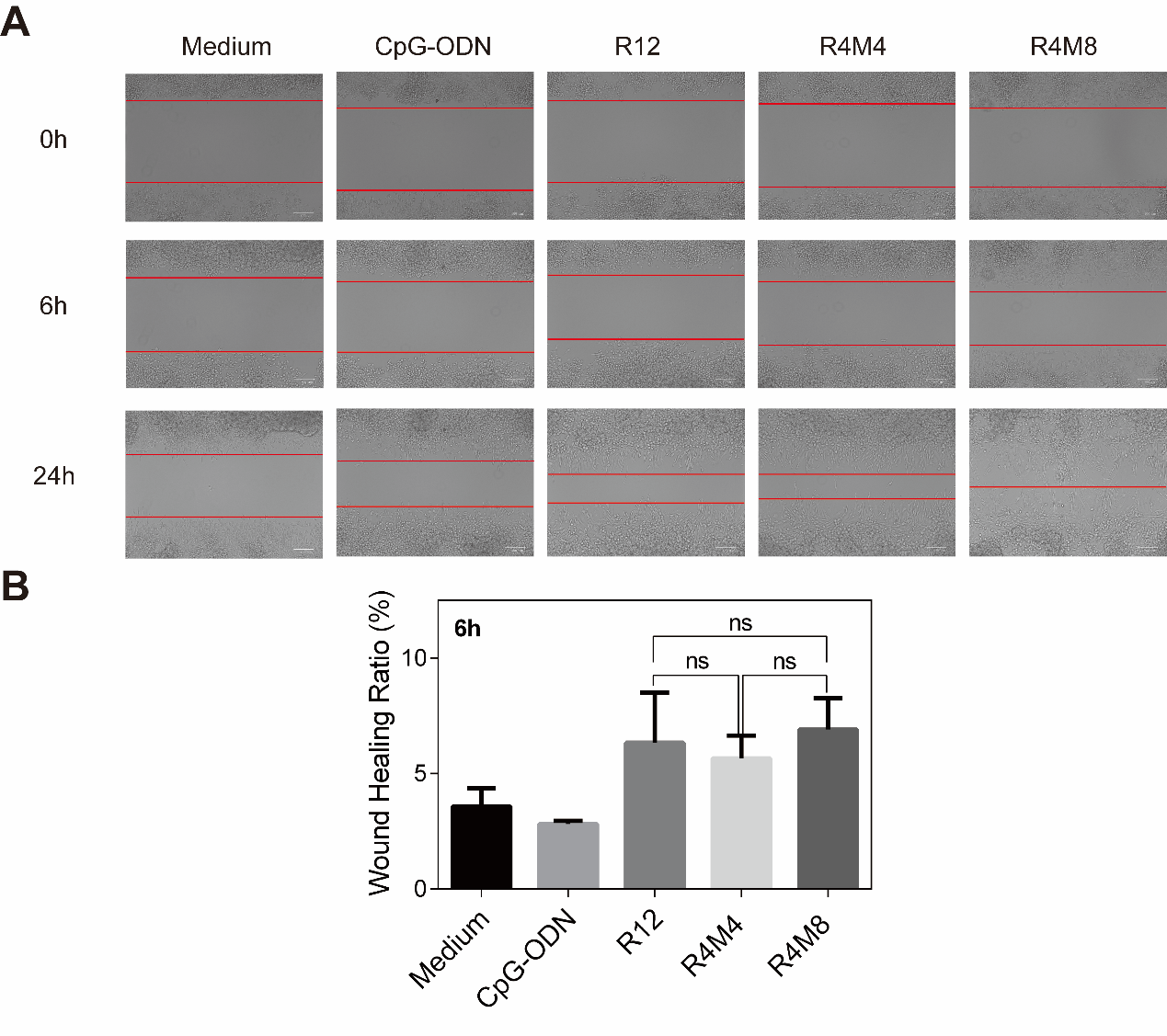


**Figure S6.** Scratch wound migration assays of CpG-ODN, CpG-RCA-gel (R12) and CpG-MCA-gels (R4M4 and R4M8) for RAW264.7 cells after incubating 6 h and 24 h. (A) The photographs of scratch wound area in 0 h, 6 h, and 24 h. (B) The wound healing ratio of gels at 6 h. CpG-MCA-gels obviously promoted the heal of wound compared to the CpG-ODN group but there is no significant difference among R12, R4M4 and R4M8 groups.
